# An Instrument to Measure Adherence to Weight Loss Programs: The Compliance Praxis Survey-Diet (COMPASS-Diet)

**DOI:** 10.3390/nu5103828

**Published:** 2013-09-26

**Authors:** Monika Janda, Doris Zeidler, Gabriela Böhm, Rudolf Schoberberger

**Affiliations:** 1 School of Public Health, Queensland University of Technology, Brisbane, Queensland, 4059, Australia; 2 Ludwig Boltzmann Institute Health Promotion Research, 1020 Vienna, Austria; E-Mail: doris.zeidler@inode.at; 3 Center of Public Health, Institute of Social Medicine, Medical University of Vienna, 1090 Wien, Austria; E-Mails: gabriela.boehm@meduniwien.ac.at (G.B.); rudolf.schoberberger@meduniwien.ac.at (R.S.)

**Keywords:** COMPASS-diet, weight-loss, surveys

## Abstract

Adherence to behavioral weight loss strategies is important for weight loss success. We aimed to examine the reliability and validity of a newly developed compliance praxis-diet (COMPASS-diet) survey with participants in a 10-week dietary intervention program. During the third of five sessions, participants of the “slim-without-diet” weight loss program (*n* = 253) completed the COMPASS-diet survey and provided data on demographic and clinical characteristics, and general self-efficacy. Group facilitators completed the COMPASS-diet-other scale estimating participants’ likely adherence from their perspective. We calculated internal consistency, convergent validity, and predictive value for objectively measured weight loss. Mean COMPASS-diet-self score was 82.4 (SD 14.2) and COMPASS-diet-other score 80.9 (SD 13.6) (possible range 12–108), with lowest scores in the normative behavior subscale. Cronbach alpha scores of the COMPASS-diet-self and -other scale were good (0.82 and 0.78, respectively). COMPASS-diet-self scores (*r* = 0.31) correlated more highly with general self-efficacy compared to COMPASS-diet-other scores (*r* = 0.04) providing evidence for validity. In multivariable analysis adjusted for age and gender, both the COMPASS-diet-self (*F* = 10.8, *p* < 0.001, *r*^2^ = 0.23) and other (*F* = 5.5, *p* < 0.001, *r*^2^ = 0.19) scales were significantly associated with weight loss achieved at program conclusion. COMPASS-diet surveys will allow group facilitators or trainers to identify patients who need additional support for optimal weight loss.

## 1. Introduction

In 2008, between 35% to 55% of women and 51% to 69% of men within European Union (EU) countries were overweight or obese [1]. Associated with excess weight are a range of chronic physiological changes commonly leading to morbidity and premature death, such as high blood pressure, high cholesterol, diabetes, and disrupted hormonal patterns, as well as musculoskeletal disorders. Lifestyle factors, including low levels of physical activity and high intake of energy dense foods and alcohol are thought to be the main contributors to this increasing burden. The EU commission therefore recommends weight loss and physical activity programs to be implemented at local, community, state and EU-wide levels [2]. Several EU member states provide weight loss programs, often funded for their members by local health care insurances [3]. In Austria, the first iteration of the 10-week “Slim-without-diet” program was developed in 1979, and has been regularly refreshed and updated according to the latest evidence [4,5]. Since 2005, it has been provided free of charge to patients interested in weight loss funded by the Lower Austria health insurance. In a recent evaluation, based on 4053 participants who enrolled between March 2005 and December 2010, within the 10-week program, participants achieved an average weight loss of −3.42 kg (range +8.0 kg to −24.0 kg), closely following the program’s recommended weight loss of 0.5 kg per week [6]. It has been shown that in contrast to more rapid weight loss slower but continuous weight loss is associated with sustained success [7]. During the subsequent six months, participants continued to lose weight (average −1.43 kg). Weight loss success was associated with the number of sessions attended in a linear fashion [6].

Given this strong association between attendance and weight loss success, it is therefore important to assess patients’ likely adherence to the program early and precisely, allowing to provide support and encouragement specifically to those participants at higher risk of ceasing the program prematurely or not following important program components. Adherence has been defined by the World Health Organization as “the extent to which a person’s behavior—taking medication, following a diet, and/or executing lifestyle changes, corresponds with agreed recommendations from a health care provider” [8]. Previously, in the setting of cardiovascular disease, we developed the compliance praxis survey (COMPASS) [9], which measures four components of treatment adherence including normative behavior (N), organizational barriers (O), social support (S) and wisdom/knowledge (W). When completed by the patients’ treating doctor, the COMPASS survey has good predictive value for the adherence to medication six months after first prescription [9]. Due to the absence of similar adherence measures in the context of weight loss, we developed two versions of the COMPASS survey to be used in the context of a weight loss program, firstly, a doctor/group facilitator instrument, COMPASS-diet-other, which estimates participants’ likely adherence from the group facilitators’ perspective and secondly, the COMPASS-diet-self instrument suitable for participant self-completion. It was the aim of this study to assess whether the COMPASS instruments could be used to determine treatment adherence within the setting of a weight loss program, establish its reliability and validity, and determine whether it was associated with actual weight loss achieved.

## 2. Methods

Approval for the present study was obtained from the University of Vienna, Center of Public Health’s, institutional review board committee.

### 2.1. The “Slim without Diet” Program

The “Slim-without-diet” program has been described in detail previously [4,5,6]. It is based on behavior modification theory and practice by Kanfer *et al.* [10], and is provided to participants within five group session plus printed self-help materials. Briefly, it provides participants with information and facts about healthy eating, the cultural, behavioral and social determinants of eating, and behaviors associated with weight management including stimulus control. It is delivered using behavioral counseling methods and trains participants in self-awareness and self-management skills [10]. It recommends slow, but steady weight loss of −0.5 kilograms (kg) per week until the participant’s target weight is reached. Participants’ height, weight and waist circumference are measured objectively at enrolment, at the end of the 10-week program and at 12 months follow-up. Regular self-weighing is recommended, and self-reported weight is collected at other time points throughout the program and at six months follow-up. The group setting facilitates social and peer support.

### 2.2. The COMPASS Survey

The COMPASS survey was developed in 2000, and measures four components of adherence “**Normative behavior**”, “**Effective structural circumstances**”, “**Social support**” und “**Wisdom/Knowledge**” (NESW) [9]. Each of these components contains three items answered on a nine-point likert scale (completely disagree to completely agree); the total score thus ranges from 12–108 points. The survey was adapted to suit the “Slim-without-diet” program aims, for example instead of querying medication adherence, the COMPASS-diet survey asks about adherence to “Slim-without-diet” program components. In addition to the doctor/group facilitator/other-rated instrument, we also developed a COMPASS-diet-self instrument suitable for participant self-completion, allowing the comparison of self- and other- rated COMPASS-diet scores. Face validity was established by cognitive interviews with five “Slim-without-diet” program participants and three group facilitators. Minor wording revisions were undertaken based on these cognitive interviews. Factor analysis resulted in a factor structure similar to the original COMPASS scale, with four factors explaining 68% of the Compass-diet-self variance, and four factors explaining 78% of the Compass-diet-other scale variance.

### 2.3. Assessment of Reliability and Validity of the New COMPASS Surveys

Participants’ demographic details including age, gender, current working status, weight at enrolment into the “Slim-without-diet” program, and comorbidity burden were assessed at baseline. Patients were asked to state their target (desired) weight, by indicating the kg value of the body weight that they would like to reach. To establish convergent validity of the newly formed COMPASS-diet instruments, participants were also asked to complete the general self-efficacy scale by Schwarzer and Jerusalem [11,12]. This scale measures general outcome expectancy beliefs using 10 items rated on a four-point likert scale (ranging from “do not agree” to “completely agree”). Summary scores range from 10 to 40, with mean score of 29 (Standard deviation (SD) 4) observed in a large sample used to establish the questionnaire’s psychometric characteristics. Reported Cronbach alpha scores for the general self-efficacy scale are excellent, and range between 0.7 and 0.9. We tested the correlation between the self-efficacy scale, and the COMPASS-diet-self and COMPASS-diet-other scores, respectively. Participants’ self-reported weight was recorded at each session, and weight was objectively measured at the beginning and the end of the 10-week “Slim-without-diet” program. If participants with higher self- or other-COMPASS scores had higher objectively measured weight loss (as well proportion of starting weight lost) at the end of the “Slim-without-diet” program, this would provide evidence for the validity of the new scales.

### 2.4. Other Measures

In addition, participants self-completed a questionnaire assessing self-reported reasons for weight gain, for example, due to pregnancy, menopause, stopping smoking or changes in life circumstance. Participants also reported on eating habits (preferred food groups, food patterns e.g., snacking), and preferred physical activities (e.g., walking, jogging, cycling) (data not used in this study).

### 2.5. Participants

Between March and September 2009, 273 participants attended an information session describing the “Slim-without-diet” program, and after a 2-week period of consideration, 253 (92.3%) participants enrolled. Written informed consent was obtained from all participants. At the third group session participants were asked to self-complete the COMPASS-diet and general self-efficacy scale. Group facilitators completed the COMPASS-diet-other estimating adherence for each participant. Completed questionnaires were available for 166 (self) and 153 (other) participants.

### 2.6. Statistical Methods

SPPS version 19 was used for all analyses. Descriptive statistics summarized participant characteristics, current and desired weight as well as actual weight loss achieved during the 10-week program. Both self- and other- rated COMPASS-diet scores were available for 106 participants, while 60 participants completed COMPASS-self only, and 47 participants had COMPASS-other scores only. For forty participants neither self- nor other-COMPASS were available. We used one-way Anova and Tukey posthoc tests to establish differences between participants’ characteristics depending on the availability of COMPASS scores. To ascertain the internal reliability of the newly developed COMPASS Scales we calculated Cronbach alpha scores. Convergent validity was assessed by computing Pearson correlation between the COMPASS-diet-self/other scores and the self-efficacy scale. To assess the predictive value of the COMPASS-diet-self/other scales we first assessed unadjusted association between objectively measured weight loss in kg, as well as proportion of starting weight lost, at the end of the “Slim-without-diet” program, participants’ and age, gender, COMPASS scores, and desired weight using Pearson correlations. We then built linear regression models adjusted for age and gender to assess whether the COMPASS-diet self rating or COMPASS-diet other rating significantly predicted actual objectively measured weight loss in kg at the end of the “Slim-without-diet” program. Other covariates such as current work were also considered for inclusion in the model, but found not to be significantly associated.

## 3. Results

Within the study time period, the “Slim-without-diet” program was attended by 203 women and 50 men. On average, participants were 47 years old (SD = 12 years), with a body mass index of 31.8 (SD =5.7). While there was no difference in weight loss by age (*p* > 0.05), the average weight loss achieved by men (5.1 kg) was higher than that of women (3.6 kg) (*p* < 0.001). Similarly, men lost a greater proportion of their starting weight (4.9%) compared to women (3.9%; *p* < 0.02). Table 1 highlights further demographic and phenotypic details of participants. Depending on the availability of COMPASS scores, participants differed, with participants without COMPASS scores being somewhat younger, more likely to be female, and currently employed. Participants without a COMPASS score lost less weight on average (1.4 kg) compared with other participants (3.9 kg) (Table 1). Subscale scores for both the COMPASS-diet-self and -other scales were highest for the Wisdom/knowledge sub-scale (mean 22.9 (SD 4.5) and mean 22.4 (SD 3.5), respectively), whereas the normative behavior subscale self- and other-scores measuring the degree of lifestyle changes and of following program recommendations were lowest (mean 18.7 (SD 4.6) and mean 19.4 (SD 4.8), respectively).

**Table 1:** Characteristics of participants by availability of adherence rating (*N* = 253).

Participant characteristics	No adherence rating (*n* = 40)	Self-adherence rating only (*n* = 60)	Other-adherence rating only (*n* = 47)	Both self and other rating (*n* = 106)	
Categorical characteristics	*N* (%)	*N* (%)	*N* (%)	*N* (%)	*p*
Gender					0.08
Women	36 (85.0)	52 (86.7)	40 (85.1)	77 (72.6)	
Men	6 (15.0)	8 (13.3)	7 (14.9)	29 (27.4)	
Working status					0.03
Employed/self-employed	31 (77.5)	28 (46.6)	23 (48.9)	70 (66.3)	
Retired/home duties	6 (15.0)	24 (40.0)	19 (40.4)	26 (24.5)	
other	3 (7.5)	8 (13.4)	5 (10.6)	10 (9.4)	
Body mass index					0.196
Normal <25 kg/m^2^	6 (15.0)	3 (5.0)	5 (10.6)	9 (8.5)	
Overweight (25–29.9 kg/m^2^)	16 (40.0)	16 (26.7)	11 (23.4)	41 (38.7)	
Obese class I (30–34.9 kg/m^2^)	13 (32.5)	22 (36.7)	20 (42.6)	30 (28.3)	
Obese class II (≥35 kg/m^2^)	5 (12.5)	19 (31.7)	11 (23.4)	26 (24.5)	
Comorbidity burden					0.61
none	39 (66.1)	33 (54.1)	29 (59.6)	64 (60.4)	
Continuous characteristics					
Current age M(SD)	41.7 (12.2)	49.8 (11.5)	50.0 (11.5)	46.9 (11.9)	0.003
Starting weight					0.45
Weight M(SD)	83.2 (13.6)	88.8 (17.6)	87.7 (21.5)	87.7 (17.2)	
Starting BMI					
BMI M(SD)	30.3 (4.9)	33.0 (5.7)	32.2 (7.0)	31.5 (5.1)	0.12
Desired weight					0.18
Weight kgM(SD)	68.4 (9.4)	71.4 (11.3)	70.0 (9.8)	72.7 (11.3)	
Weight loss achieved					<0.001
Weight kgM(SD)	1.4 (1.8)	3.3 (2.5)	3.8 (2.7)	4.8 (3.0)	
Relative weight loss					<0.001
% of starting weight lost	1.7 (2.1)	3.7 (2.7)	4.3 (2.7)	5.3 (3.0)	
COMPASS-diet (self)					0.47
Summary Score M(SD)		81.6 (15.1)		83.2 (13.3)	
COMPASS-diet (other)					0.09
Summary Score M(SD)			78.9 (15.4)	82.9 (11.9)	
General self-efficacy					
Summary Score M(SD)		32.1 (3.6)		31.8 (3.9)	0.64

Both the COMPASS-diet-self (Cronbach α = 0.82) and the COMPASS-diet-other scales (Cronbach α = 0.78) had good internal reliability (Cronbach α scores for the subscales were acceptable and ranged from 0.52 to 0.88, except for the COMPASS-diet-self social support subscale, which had a low score of 0.44). Participants with COMPASS-diet-self scores below the mean lost less weight (−3.1 kg) compared to participants with high scores (−4.9 kg; *p* < 0.006). The association between higher COMPASS-diet-other scores and actual weight loss was also in the expected direction (−3.9 kg in participants with COMPASS-diet other scores below the mean, compared to −5.1 kg in those with scores above the mean; *p* < 0.02). The correlation between participants’ general self-efficacy and their COMPASS-diet-self scores was moderate at *r* = 0.31, while the correlation between the self-efficacy score and the COMPASS-diet-other score was low at *r* = 0.04. With regards to predictive validity, the COMPASS-diet-self scores correlated moderately highly with the COMPASS-diet-other scores (*r* = 0.37), as well as the actual weight loss achieved (*r* = 0.28). The correlation between the COMPASS-diet-other score and the actual weight loss was also moderately high (*r* = 0.28, *p* < 0.001)). The highest correlation was observed between participants desired weight at program start and actual weight loss achieved (*r* = 0.44) (Table 2). Correlations were of similar magnitude when considering participants’ weight loss relative to the starting weight (*r* = 0.26 with COMPASS-diet-self and *r* = 0.30 with COMPASS-diet-other).

**Table 2:** Pearson correlations between participants age, self-efficacy, self or other rated compliance praxis-diet (COMPASS-diet) score, desired and actual weight loss.

	Age	General self-efficacy	COMPASS-diet self	COMPASS-diet other	Desired weight at start of program
Age	1				
General self-efficacy	0.16 *	1			
COMPASS-diet self	0.08	0.31 **	1		
COMPASS-diet other	−0.17 *	0.04	0.37 **	1	
Desired weight	0.13	0.15	0.14	−0.01	1
Actual weight loss achieved	−0.01	0.07	0.28 **	0.28 **	0.44 **

* *p* < 0.05; ** *p* < 0.001.

For participants who completed the COMPASS-diet-self scale, in multiple linear regression analysis mutually adjusted for all other factors, being male, of younger age and having a higher COMPASS-diet-self score were predictive of higher weight loss at the end of the program. For participants with a COMPASS-diet-other score, male gender, and a higher COMPASS score predicted weight loss success (Table 3).

**Table 3:** Multiple linear regression analyses examining association between baseline characteristics, self (*n* = 166—Model 1) or other (*n* = 153—Model 2) rated COMPASS-diet score and weight loss achieved at the end of the intervention program.

	Model 1	Model 2
Gender	0.37 ***	0.39 ***
*Age*	−0.15 *	−0.08
General self-efficacy	−0.05	−0.04
COMPASS-Self	0.31 ***	-
COMPASS-Other	-	0.22 *
**Total *R*^2^**	0.23	0.19
**Total Adjusted *R*^2^**	0.21	0.16
***F***	10.85 ***	5.51 ***

* *p* < 0.05. ** *p* < 0.01. *** *p* < 0.001. Note. (ref) indicates reference category. β = standardised regression coefficients.

## 4. Discussion

The success of weight loss programs depends on the uptake by participants of program components and active adherence to the recommended dietary and behavioral strategies, rather than on the specific dietary prescription [13]. Adherence requires participants to make an active decision for or against attending program sessions, and requires effort while putting into practice behavior change, and overcoming ingrained habits of overeating [7,8,14,15]. On average, participants of the “Slim-without-diet” program have been shown to achieve good initial, as well as persistent, weight loss up to 12 months post program completion, with success linearly associated with program session attendance [6]. Results of the present study indicate that both the COMPASS-diet-self and -other surveys could provide useful early information which could prospectively predict program success. Participants with higher COMPASS-diet-self or -other scores lost more weight than those with lower scores, even when adjusted for gender, age and general self-efficacy scores. Assessment of COMPASS-diet scores early in the program could therefore provide assistance to group facilitators to identify participants who may require additional support to achieve weight loss according to program aims. This additional support could specifically target those areas of the COMPASS diet instrument which contributed to an individual’s low score, for example address ingrained behavioral patterns, make suggestions for how to rally additional social support, or provide education to address knowledge gaps [14,16].

Previous work has described common barriers to weight loss including lack of knowledge and self-management skills, lack of time for example to cook a special diet, low social support by friends and family, socio-economic difficulties and alternative health problems requiring attention [3,17,18,19,20]. The “Slim-without-diet” program addresses all of these common barriers and how to overcome these in its program sessions, with a specific focus on self-monitoring. Self-monitoring has been consistently associated with greater weight loss in a number of previous studies, probably reflecting the overall motivation and skills of the participants [21]. Results from the COMPASS survey could enable participants to identify the skills they are lacking and specifically focus some of their self-monitoring effort on these aspects of weight management. Several novel mobile phone applications have recently been developed assisting users with self-management or social support skills, and could be useful especially for program participants with low COMPASS scores [16,22,23].

COMPASS-diet subscale scores highlighted that it is easier for the majority of participants to acquire the necessary knowledge than change ingrained behavior and follow through with the suggested behavioral changes. This is similar to previous research, which showed that knowledge is a necessary but not sufficient component of behavior change [24]. The “Slim-without-diet” program has a number of components which aim to overcome resistance to behavior change including providing peer support, modeling successful weight loss behaviors and training in self-reward. Additional support that could be helpful for participants especially if provided by their doctor or health care professional includes regular monitoring of body mass index, and adding discussion about the benefits of a healthy weight and sufficient physical activity to consultations. Regular reinforcement by trusted health professionals has been shown to positively influence weight loss efforts [25,26,27]. However studies have also evidenced, that such opportunity is only used in less than half of the clinic visits, and people are not regularly enough counseled about weight loss or at least referred for dietary and physical activity support [22,28,29]. The COMPASS survey results can assist group facilitators to identify those participants most in need for that support early.

## Limitations of This Study

While the present study provides good initial support for the psychometric properties of the newly developed COMPASS-diet surveys, their value should be confirmed in an independent prospective sample, as well as in participants who undergo other than the “Slim without diet” programs. The present sample contained a larger proportion of women than men, and all participants received the program free of charge supplemented by their healthcare insurance. Data was unavailable for 40 participants, who were younger females, and more likely to be employed, possibly highlighting another group in need of additional support early on in weight loss programs.

## 5. Conclusions

In summary, the newly developed COMPASS-diet surveys could be a useful addition to the assessment portfolio of people who enroll into a weight loss program besides currently available measurements scales, providing an early indication of their likely adherence and weight loss success.
